# Supplementary figures and images for: Ehrlichia SLiM Ligand Mimetic Activates Notch Signaling in Human Monocytes
Source: mBio. 2022 Mar 31;13(2):e00076-22. doi: 10.1128/mbio.00076-22 (PMC9040721; doi:10.1128/mbio.00076-22)

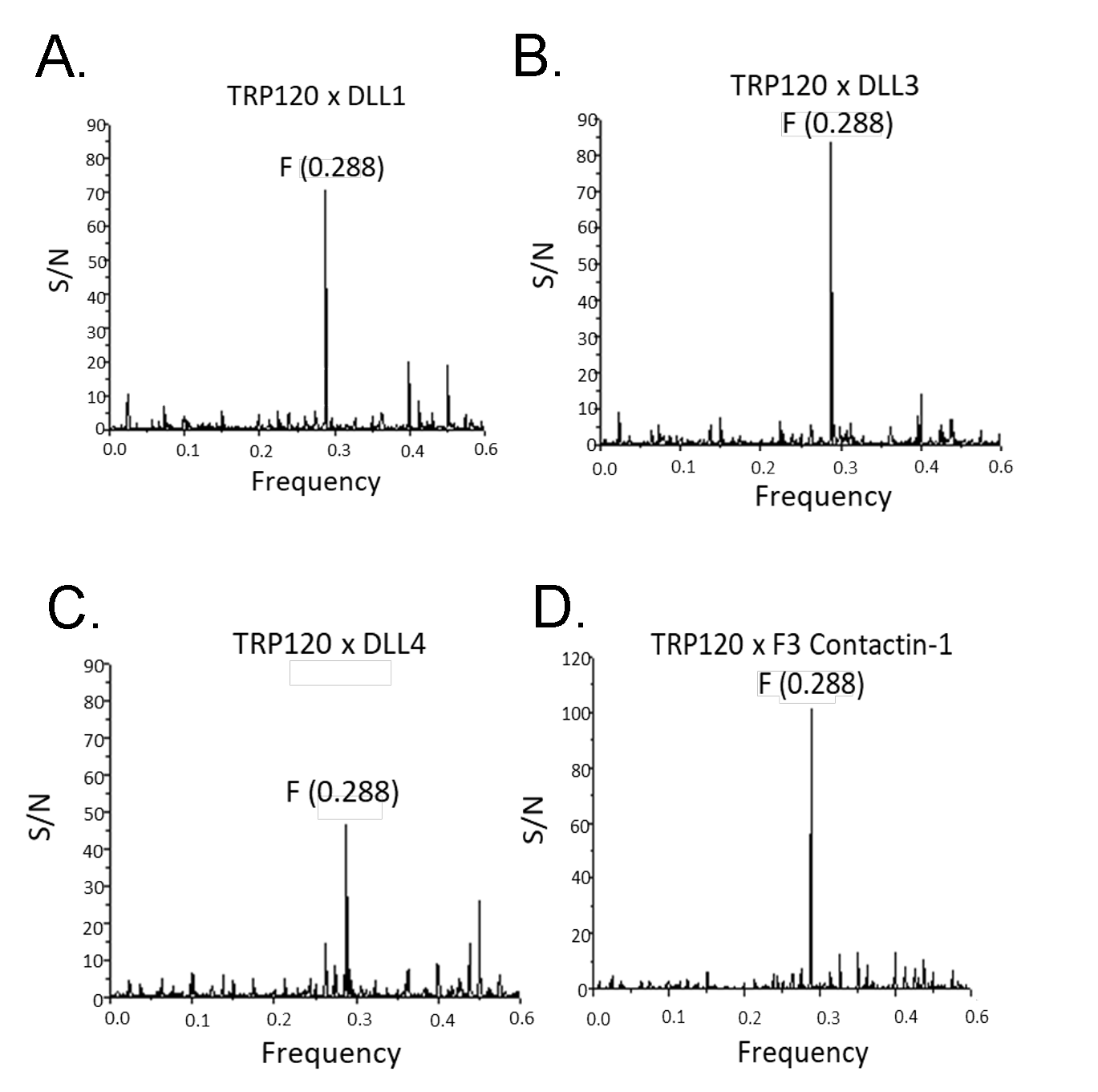

Supplement: FIG S1 [file mbio.00076-22-sf001.tif]

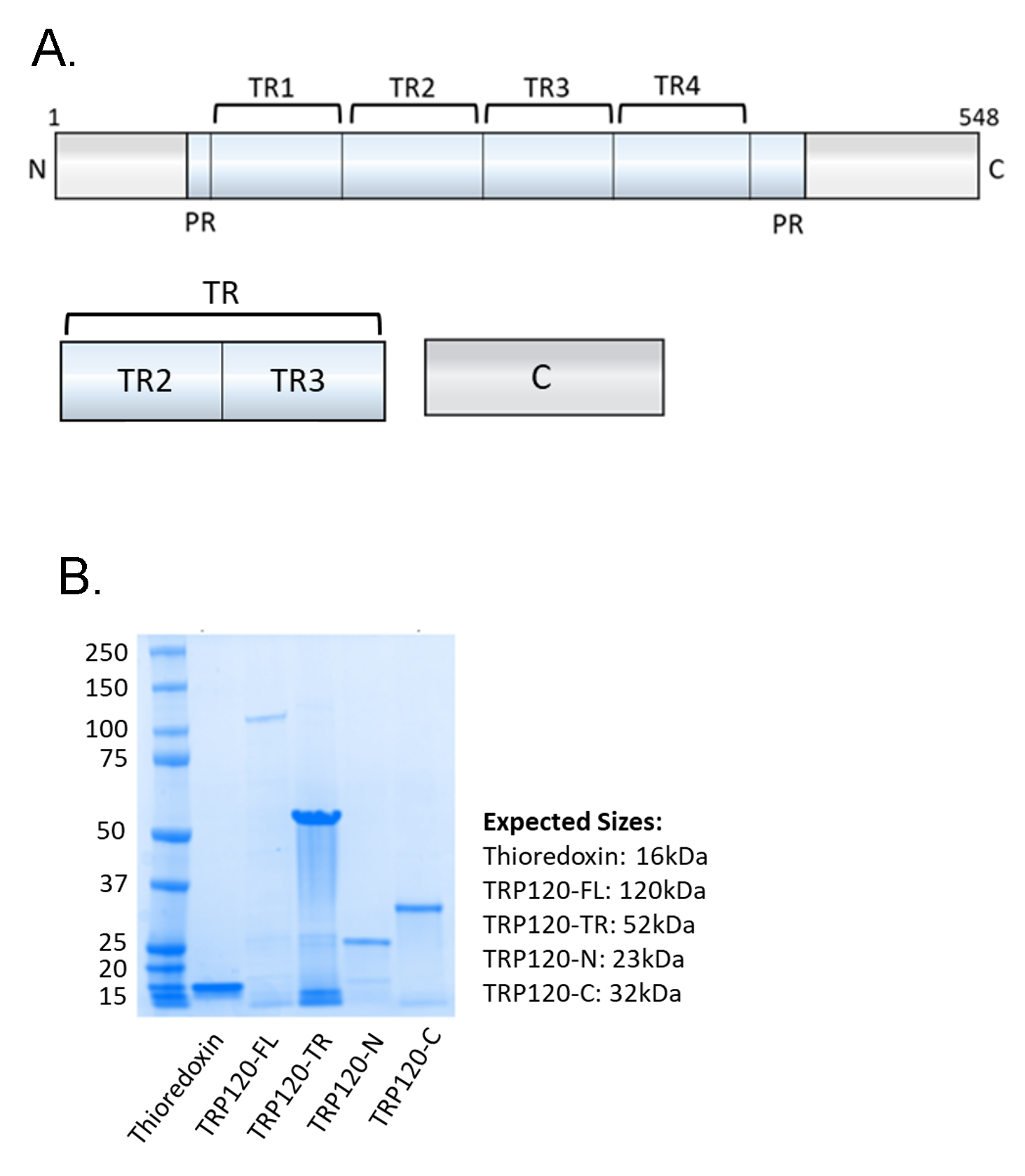

Supplement: FIG S2 [file mbio.00076-22-sf002.tif]

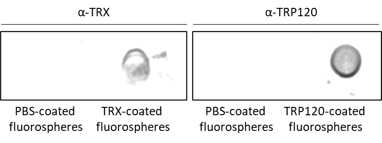

Supplement: FIG S3 [file mbio.00076-22-sf003.tif]

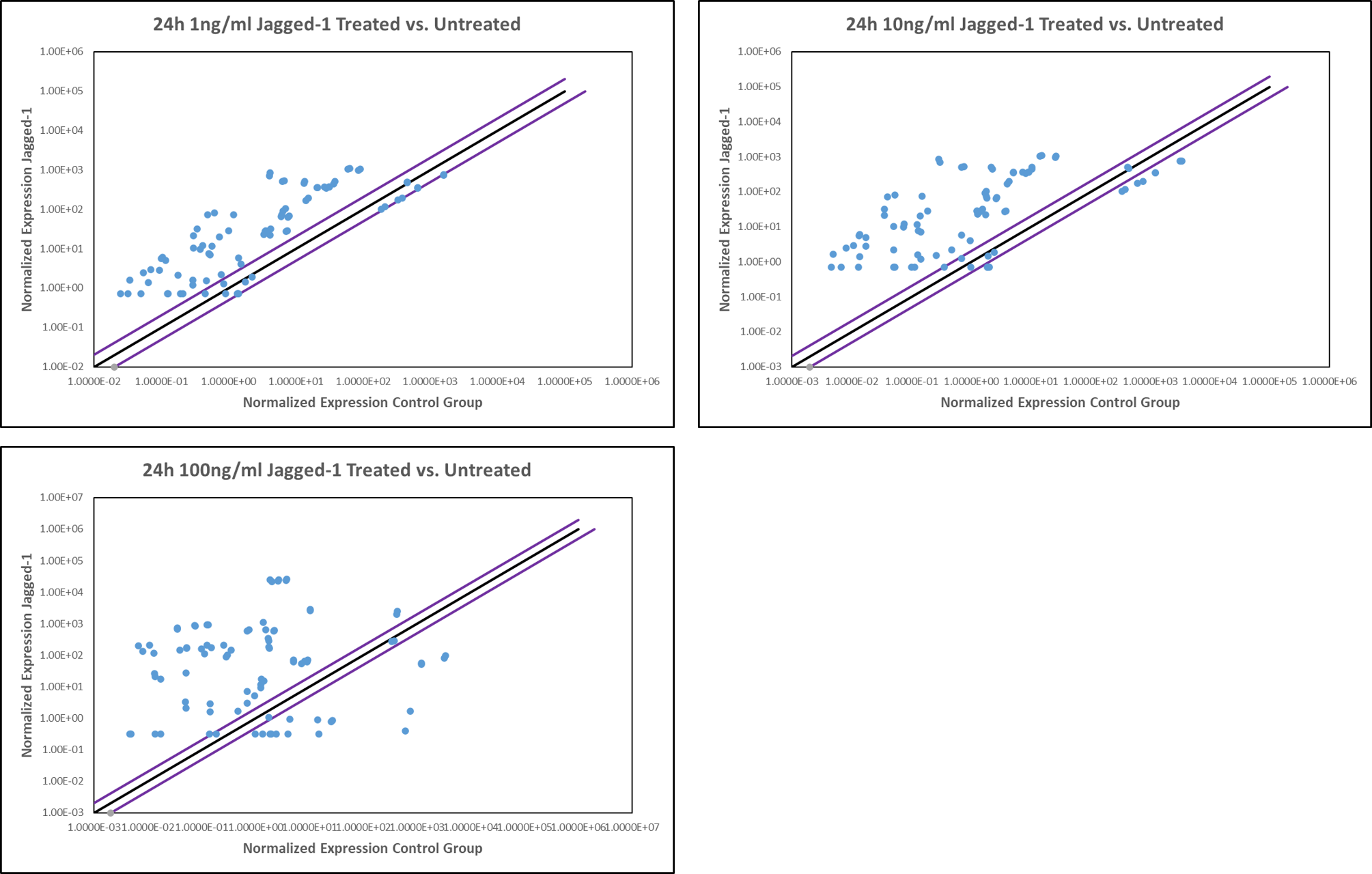

Supplement: FIG S4 [file mbio.00076-22-sf004.tif]
